# Age- and disease-related features of task-related brain oscillations by using mutual information

**DOI:** 10.1002/brb3.93

**Published:** 2012-09-23

**Authors:** Chia-Ju Liu, Chin-Fei Huang, Chia-Yi Chou, Wen-jin Kuo, Yu-Te Lin, Chao-Ming Hung, Tsung-Ching Chen, Ming-Chung Ho

**Affiliations:** 1 Graduate Institute of Science Education, National Kaohsiung Normal UniversityKaohsiung, Taiwan; 2 Department of Environmental and Occupational Safety and Hygiene, Graduate Institute of Environmental Management, Tajen UniversityPingtung, Taiwan; 3 Department of Physics, National Kaohsiung Normal UniversityKaohsiung, Taiwan; 4 Section of Neurology, Kaohsiung Veterans General HospitalKaohsiung, Taiwan; 5 Section of General Surgery, E-Da HospitalKaohsiung, Taiwan

**Keywords:** Aging, cross-mutual information, electroencephalogram, mild cognitive impairment, theta band

## Abstract

The aim of this study was to investigate changes in task-related brain oscillations and corticocortical connections in patients with mild cognitive impairment (MCI) and those with normal aging using cross-mutual information (CMI) analysis. We hypothesized that task-related brain oscillations and corticocortical connections were affected by age- and disease-related changes, which could be reflected in the CMI analysis. Electroencephalogram (EEG) recordings were measured in 16 MCI patients, 15 healthy age-matched controls, and 16 healthy younger individuals. The frequencies and interhemispheric CMI data were estimated in all groups. The specific EEG rhythms measured were delta (δ), theta (θ), alpha (α), beta (β), and gamma (γ) bands. Significant differences in δ, θ, α, and β bands were observed between the younger and elderly groups. However, only the θ band was significantly different between the elderly and MCI groups. Moreover, this study used EEG recordings to investigate age- and disease-related changes in the corticocortical connections of the brain. This study proves that the θ-band frequency of the connection between the parietal and occipital lobes for the age- and disease-related changes can be depicted using the CMI analysis.

## Introduction

It is still unclear to differentiate mild cognitive impairment (MCI) from normal brain aging. MCI is defined as cognitive impairments beyond what is expected from normal aging (Sakuma et al. 2007). The interest and importance of MCI are growing as subjects with MCI have a high annual conversion rate to dementia (Petersen et al. 2001; Frodl et al. 2002; Babiloni et al. 2010). As a result, it is necessary to have a better understanding of the differences between normal aging and MCI (Chapman et al. 2009). In past studies, electroencephalogram (EEG) recordings and functional magnetic resonance imaging (fMRI) have been widely used to investigate the changes in brain activity associated with age- and disease-related features (Jelic et al. 2000). However, the nature of task-related brain oscillations in healthy aging and MCI disease-related features remains poorly understood (Bassett and Bullmore 2009; Phillips and Andrés 2010; Ho et al. 2012).

Many studies have addressed the reliable and sensitive components of event-related potentials (ERPs) when exploring the changes between age- and disease-related features (Stam 2005; Chapman et al. 2009; Lai et al. 2010). Nevertheless, it is difficult to evaluate the corticocortical connections by ERP analysis. To overcome this difficulty, this study used cross-mutual information (CMI) quantification of task-related EEG data to reflect the different connections of information processing in the brain. Because the quantification of the task-related EEG recordings may include linear and nonlinear characteristics, it is appropriate for this study to use the mutual information (MI) method, which detects statistical dependencies among time series (Jeong et al. 2001; Na et al. 2002; Wang et al. 2009; Jin et al. 2010). Additionally, the CMI method quantifies information transmitted from one electrode to another, and it is good at assessing the strength of synchronization among areas that reflected precisely the coupling of these areas by corticocortical connections (Singer 1999; Jeong et al. 2001; Jin et al. 2010).

Broadband filtered data have been used to calculate the CMI in the multichannel EEG recordings of participants. In previous studies, theta (θ) and beta (β) frequency bands were analyzed to explore the differences between healthy participants and patients with MCI (Kowalski et al. 2001; Pijnenburga et al. 2004; Jelles et al. 2008). However, the use of delta (δ) and alpha (α) frequency bands to investigate the changes between MCI and normal aging is still under debate (Kowalski et al. 2001; Babiloni et al. 2006). Kowalski et al. (2001) demonstrated that there is no relationship between the presence of δ waves and the level of cognitive impairment. On the contrary, Babiloni et al. (2006) implied that δ waves may be correlated with MCI disease-related features. Therefore, this study aimed to clarify which frequency bands and corticocortical connections of task-related brain oscillations reflect the age- and MCI disease-related changes using CMI analysis.

The past study of spontaneous brain oscillatory activity in a resting state has provided useful information for aging- and disease-related research, yet a more comprehensive explanation of real-life evocation of brain oscillations is needed in ERP studies (Basar and Guntekin 2008). Greater attention is being paid to evoke oscillatory activity in the brain while performing cognitive tasks in healthy aging people and people with disease-related changes (Phillips and Andrés 2010). Hence, this study focuses on task-related brain oscillations to investigate the age- and disease-related features.

We hypothesized that task-related brain oscillations and corticocortical connections are affected by age- and disease-related changes, which can be reflected with the CMI analysis. To our knowledge, this study represents the first study of brain oscillation in participants with age- and disease-related changes using the CMI analysis of task-related brain oscillations.

## Materials and Methods

### Participants

This study recruited three groups of participants: one consisting of 16 younger individuals (mean age ± SD: 22.5 ± 2.7 years; 6 female and 10 male), another of 15 elderly people (mean age ± SD: 70.1 ± 8.5 years; 5 female and 10 male), and the other of 16 MCI patients (mean age ± SD: 79.3 ± 8.5 years; 6 female and 10 male). All participants underwent the Chinese version of the Mini-Mental Status Examination (MMSE) (Folstein et al. 1975). The MCI group had a mean MMSE score of 25.4 ± 2.1 (possible range 0–30), while the elderly group had a mean MMSE score of 29.2 ± 0.7 with a score of 30 for the younger group. The MCI patients were diagnosed at the Kaohsiung Veterans General Hospital, Taiwan. All MCI patients fulfilled the criteria described by Petersen et al. (1999) for MCI. These criteria have been used in previous studies (Frodl et al. 2002; Pijnenburga et al. 2004; Babiloni et al. 2006), which contain (a) objective memory impairment, (b) normal activities of daily living, (c) normal general cognitive function, and (d) memory deficits beyond what is expected for their age through the clinical diagnosis by the specialized physician. In addition, patients with mild AD and concomitant dementia were excluded from the MCI group. The differences between healthy elderly group and MCI group provide the information about disease-related changes, while those between healthy elderly group and younger group provide the information about age-related changes. None of the participants reported hearing loss or psychological diseases. No one suffered from high blood pressure (BP), diabetes, heart disease, and all were naïve to the electrophysiological studies. Written informed consent was obtained from all participants before conducting this experiment. This study conformed to the Code of Ethics of the World Medical Association (Declaration of Helsinki), and the protocol of this study was approved by the ethics committee of the National Kaohsiung Normal University.

### Materials

**Figure 1:**
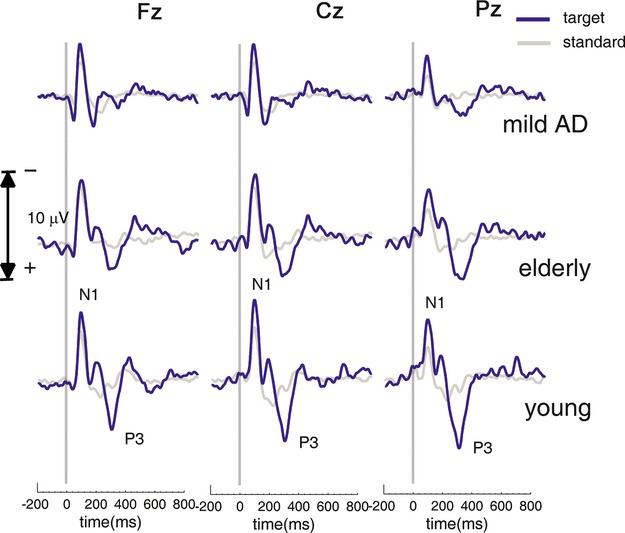
ERP time series over the entire epoch.

**Figure 2:**
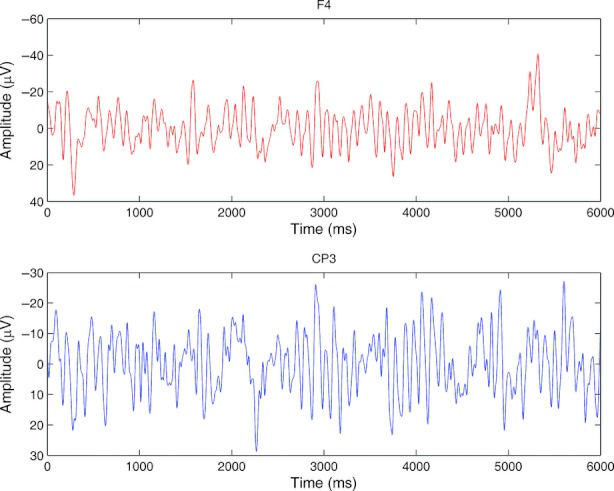
Two examples (F4, CP3) of the EEG data, which were segmented into 6000-msec epochs. Each epoch included three trials and the triggers onset at 0, 2000, and 4000 msec.

The oddball stimulus paradigm was used to elicit auditory ERPs; this test requires little time and is a simple task for MCI patients (Goodin 2005). The EEG was amplified and filtered (band pass, 0.01–50 Hz, 12 dB/octave, and zero phase shift) by the SynAmps/SCAN 4.4 hardware and software (NeuroScan, Inc., Herndon, VA), using the commercial Electro-Cap (Electro-Cap International, Eaton, OH) with electrodes placed at 30 scalp locations (FP1, FP2, F7, F8, F3, F4, FZ, FT7, FC3, FCZ, FC4, FT8, T3, C3, CZ, C4, T4, TP7, CP3, CPZ, CP4, TP8, PZ, P3, P4, T5, T6, O1, OZ, O2) based on the 10–20 system. The reference electrodes were placed in the earlobes. The electrode impedance was maintained below 5 kΩ. Stimulus presentation was generated by Neuroscan Stim 3.3 software. EEG channels were continuously digitized by a SynAmp amplifier. The signal was analog filtered and A/D converted with a sampling rate of 1000 Hz and 14-bit precision. The auditory oddball task was elicited with pure tones including 1000- and 2000-Hz frequencies. The standard (2000-Hz frequency) and target (1000-Hz frequency) auditory stimuli were presented binaurally over headphones to each participant at a sound pressure level (SPL) of 85 dB with a duration of 20 msec. The rise and fall times were both 10 msec with a 2-sec interstimulus interval. The ratio of target to standard stimuli was 1:4 and presented randomly. This experiment consisted of two blocks with 300 trials in each block. All participants were required to distinguish between the two pure tones and press a button with the thumb of the right hand in response to the target stimulus but not the standard stimulus. Electrooculogram (EOG) and muscular artifacts were rejected by the Neuroscan software, with only traces lower than 70-μV peak-to-peak being accepted. Before computing the EEG data by CMI, the ERP data were analyzed (Fig. 1). The EEG data were segmented into 6000-msec epochs (Fig. 2). Each epoch included three trials. Sweeps exceeding ±70 μV were excluded by automatic artifact screening. MATLAB 7.1 and EEGLAB (Delorme and Makeig 2004) software were used to apply a phase-corrected FIR filter in the δ (1–4 Hz), θ (4–7 Hz), α (7–13 Hz), β (13–25 Hz), and γ (25–50 Hz) frequency bands.

### CMI analysis

This study analyzed task-related brain oscillations using CMI analysis. CMI quantifies the information transmitted from one electrode to another (Jeong et al. 2001). The CMI analysis was defined as (Jeong et al. 2001):


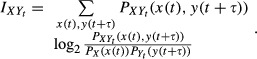


This study evaluated the probabilities by constructing a histogram (from 6000 data points) of the variations of the measurement. The CMI term, 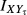, is between time serials data *x*(*t*) and *y*(*t*+*τ*). The τ of the *y* function is time delayed. *P*_*X*_ (*x*(*t*)), 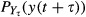, and 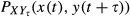 represent the normalized histogram of the distribution of values observed for the measurement *x*(*t*) and *y*(*t* + τ). The sampling frequency was 1000 Hz, and the time delay of the CMI was normalized to log_2_ (bins). In this study, 36 bins were used to construct the histograms, which provide stable estimates. The average time-delayed CMI between all electrodes (over time delays of 0–500 msec) was computed to be the information transmission between different cortical areas. The CMI analysis quantified how much information was shared between two signals and the decay in the range [0–1]. As Jeong et al. (2001) mentioned:

If the measurement of a value from X resulting in *x*_*i*_ is completely independent of the measurement of a value from Y resulting in *y*_*j*_, then *P*_*xy*_(*x*,*y*) factorizes: *P*_*xy*_(*x*,*y*) = *P*_*x*_(*x*)*P*_*y*_(*y*) and the amount of information between the measurements, the MI is zero. One of the properties of the MI is that *I*_*xy*_ = *I*_*yx*_.

Based on this theory, 30 electrodes were analyzed from all participants using CMI. This study evaluated the mean CMI values between all paired electrodes. (For example, for region FP1, the mean CMI values were calculated for the following paired electrodes: FP1–F7, FP1–FP2, FP1–F3, FP1–FZ, FP1–F4, FP1–F8, FP1–FC3, FP1–FCZ, FP1–FC4.) The MI data from local regions and central lines were also estimated between all pairs of interhemispheric electrodes. The CMI analysis was performed for the following frequency bands: δ band (1–4 Hz), θ band (4–7 Hz), α band (7–13 Hz), β band (13–25 Hz), and γ band (25–50 Hz). Results were averaged over all subjects within each group and all possible electrode pairs.

The group differences of each CMI were analyzed using the analysis of variance (ANOVA) with a group factor (younger vs. elderly vs. MCI patient) and within participants factor (frequency bands). Post hoc group comparisons of mean CMI were performed using Scheffe's post hoc test (SPSS version 12.0). A two-tailed *P*-value of less than 0.05 was considered significant.

## Results

Clinical characteristics (age, sex, and MMSE scores) among different groups are shown in Table 1. The younger participants were significantly younger than the elderly and MCI groups, but there was no statistical difference between elderly and MCI groups with respect to age. Mean MMSE scores were not significantly different between the younger and elderly groups. However, compared with the MCI groups, the younger and elderly groups had significantly better MMSE scores.

**Table 1:** Clinical characteristics and examples of average values and standard deviations from the CMI data (electrodes: CP3–F4) among the younger, elderly, and MCI groups

Frequency bands	Younger group (*n* = 16)	Elderly group (*n* = 15)	MCI group (*n* = 16)	Significance (*P-*value)
Age (years)	22.5 ± 2.7^a/c^	70.1 ± 8.5^a/b^	79.3 ± 8.5^b/c^	<0.001^a^
				>0.05^b^
				<0.001^c^
Sex (m/f)	10/6	10/5	10/6	
MMSE (score)	30^a/c^	29.2 ± 0.7^a/b^	25.4 ± 2.1^b/c^	>0.05^a^
				<0.001^b^
				<0.001^c^
δ (1–4 Hz)	0.33 ± 0.03^a^	0.28 ± 0.04^a/b^	0.28 ± 0.02^b^	<0.001^a^
				>0.05^b^
θ (4–7 Hz)	0.23 ± 0.02^a^	0.20 ± 0.02^a/b^	0.16 ± 0.02^b^	<0.05^a^
				<0.001^b^
α (7–13 Hz)	0.19 ± 0.04^a^	0.15 ± 0.04^a/b^	0.12 ± 0.02^b^	<0.001^a^
				>0.05^b^
β (13–25 Hz)	0.12 ± 0.03^a^	0.10 ± 0.01^a/b^	0.09 ± 0.02^b^	<0.05^a^
				>0.05^b^
γ (25–50 Hz)	0.09 ± 0.03^a^	0.07 ± 0.02^a/b^	0.06 ± 0.02^b^	>0.05^a^
				>0.05^b^

MCI, mild cognitive impairment; m, male; f, female.

Letters a, b, and c indicate significant differences between groups using Scheffe's ANOVA post hoc test.

For the CMI analysis, the synchronization between the CP3–F4 electrodes (both long-range and interhemispheric connections) was used as an example to show representative results (Table 1). CMI data analyzed with ANOVA revealed significant main effects among the groups in the δ band (*F*_2, 44_ = 13.01; *P* < 0.001), θ band (*F*_2, 44_ = 29.75; *P* < 0.001), β band (*F*_2, 44_ = 7.25; *P* < 0.01), α band (*F*_2, 44_ = 11.86; *P* < 0.001), and γ band (*F*_2, 44_ = 4.91; *P* < 0.05). There were significant differences in all frequencies between the younger and MCI groups. However, it is difficult to explore whether this change in frequencies is due to age-related or MCI disease-related features. Table 1 presents the post hoc comparisons between the younger and elderly groups, and the elderly and MCI patients groups to further clarify which frequency bands of task-related brain oscillations could reflect the changes between age- and MCI disease-related changes using CMI analysis.

Compared with the elderly group, the younger group revealed significantly higher CMI data in the δ, θ, α, and β bands, but did not reveal significant differences in the γ band. In contrast, only the θ band was significantly different between the elderly and MCI groups.

**Figure 3:**
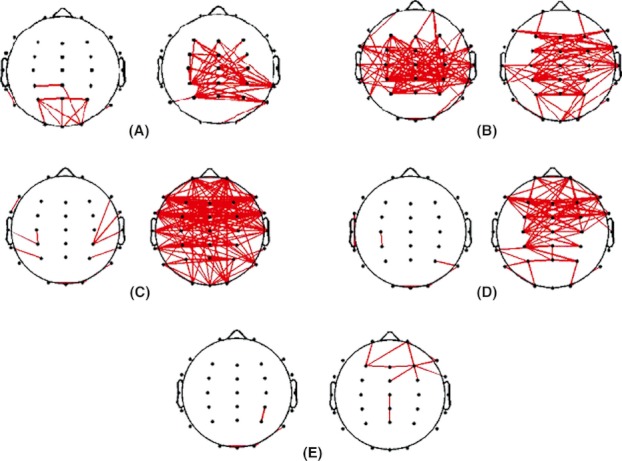
The topographic map describing all of the electrode pairs showing significant differences (unpaired Student's *t*-test, *P* < 0.05) between two compared groups. The left topographic map of each figure indicates the comparison between the elderly and MCI groups. The right topographic map of each figure indicates the comparison between the elderly and younger groups. (A) δ band; (B) θ band; (C) α band; (D) β band; (E) γ band.

In Figure 3, the CMI data are represented by red lines connecting the two paired electrodes that showed a significant effect. In other words, Figure 3 shows the topographic map describing the electrode pairs between which significant differences in CMI values (*P* < 0.05) were found. When an electrode pair revealed significant differences in CMI values, a red line will show between the two electrodes of this pair. Statistical analyses showed significant differences in the CMI of the δ band between the elderly and younger groups among the frontal, fronto-central, central, centroparietal, and parietal electrodes (e.g., F3–CP3, FC3–FCZ, FC3–CZ, CP3–CP4, CP3–P3, P3–FZ; Fig. 3A). However, significant differences in the δ band between the elderly and MCI groups were only observed between the parietal and occipital electrodes (e.g., P3–O1, PZ–P4, PZ–O2); no statistical differences among frontal pole, frontal, central, and frontocentral electrodes were found (Fig. 3A). Except in the occipital lobe, all electrodes showed significant differences between the elderly and MCI groups in the θ band (Fig. 3B). Significant differences were also found between the elderly and younger groups as shown in Figure 3B, whereas the pairs of electrodes included the occipital lobe. In the α band, there were significant differences between the elderly and younger groups in all pairs of electrodes (Fig. 3C), but significant differences in the α band between elderly and MCI groups were only observed in F7–T3, C3–CP3, CP3–TP7, P3–T5, CP4–F8, CP4–T4, CP4–TP8, and P4–T6 electrode pairs (Fig. 3C). Significant differences in the β band were found between the elderly and younger groups among the frontal pole, frontal, central, frontocentral, and centroparietal electrodes. However, the significant differences in the β band between the elderly and MCI groups were only in the T3–TP7, C3–CP3, and P4–T6 electrode pairs. Finally, in the γ band, significant differences were discovered between elderly and younger groups in the F3–FP1, F3–FP2, F3–FZ, F4–F8, F4–FP2, F4–FC4, F4–FCZ, and FCZ–PZ electrode pairs. The only significant difference in the γ band between elderly and MCI groups was found in the CP4–P4 electrode pair.

Oscillations in θ band change during attention focusing (Sauseng et al. 2008), while the phase coupling in θ oscillation is known to reflect memory-related processes (Schack et al. 2002). In addition, the long-range coupling between oscillators of θ activities has also been interpreted as indicating integration of cortical information underlying cognitive processing in the brain (Sauseng et al. 2007), and increased attention has been associated with frontal–posterior coherence of θ oscillations (Aftanas and Golocheikine 2001).

**Figure 4:**
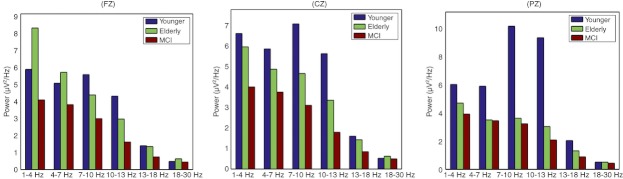
The power value in the frequency bands.

The power values were analyzed to explore the changes of brain oscillation between groups responding to the target stimuli in the various bands (Fig. 4), which demonstrated that power was higher in the young group than in the elderly group in the parietal.

In conclusion, the analysis of the topographic map indicated that the corticocortical connections which were both affected by age- and disease-related changes were reflected in θ band.

## Discussion

This study showed that frequency bands including δ, θ, α, and β bands reflect the differences between the younger and elderly groups, and it is the θ band that reflects the differences between the elderly and MCI groups. Besides, only θ bands were able to reflect the differences among the younger, elderly, and MCI groups. As a result, only the θ band by CMI analysis was affected both by age- and disease-related changes.

The results of this study confirm some previous studies but stand in contrast to others. Some studies on coherence (e.g., Besthorn et al. 1994) have demonstrated that the δ band could not reflect the difference between elderly people and MCI patience, but the other research (Huang et al. 2000) obtained adverse findings. In this study, we found no significant difference in the CMI data of the δ band between elderly and MCI groups, which supports the results found in the previous study (Kowalski et al. 2001).

Additionally, past studies have suggested that the δ band of the frontal lobe could discriminate between MCI patients and healthy elders (Penttilä et al. 1985; Coben et al. 1990; Schreiter-Gasser et al. 1993); however, the topographic map generated from this study showed that only the electrodes near the parietal and occipital lobes displayed significant differences between the elderly and MCI groups in the δ band. These inconsistent findings might be caused by the different conditions under which the data were collected and the authors' definition of the δ band. First, in this study, we analyzed task-related brain oscillations by using mutual information while a resting awake condition was applied in the previous studies. The greater attention paid to respond to the task may cause the different oscillatory activity in the brain of the participant (Phillips and Andrés 2010). In the previous study under resting conditions, Stam et al. (2003) showed that loss of β-band synchronization occurs early in mildly affected AD patients and correlates with cognitive impairment. In our study, the older group with mild AD had significantly decreased functional connectivity of the brain when attempting to respond to the target stimuli, especially in θ band, providing further evidence that as disease progresses there is less efficient processing (Grady 1998). These disease-related deficits in efficiency correlated with the reduced connections in our study, suggesting severe impairment in the information processing ability. This would explain this group's longer mean reaction time and a lower correct response.

Furthermore, many studies have suggested the importance of the θ band for discriminating among normal elders, MCI, and AD patients (Kowalski et al. 2001; Osipova 2003; Pijnenburga et al. 2004), and this was supported by the results of this study. Additionally, this study suggested that θ band reflects not only the difference in disease-related changes but also the difference in age-related changes, especially in the parietal and occipital lobe.

Some studies have also indicated that the α band was significantly different between normal elderly and AD patients in power (Hogan et al. 2003; Moretti 2004) and in connectivity (Pijnenburga et al. 2004). Unexpectedly, we did not find any statistically significant differences in the α band between the elderly group and the MCI group. Oppositely, the α band was found to be different between the young and elderly groups almost in the whole brain. There are two possible reasons for these different results. First, we analyzed task-related brain oscillations by using mutual information in this study. Compared with the previous studies with a resting awake condition, the attention paid to respond to the task under a task-related condition in this study may cause the different oscillatory activity in the brain (Phillips and Andrés 2010). Second, the different definition of α frequency band might be another reason for the inconsistent findings between this study and some past studies. Some studies (Hogan et al. 2003) defined the lower α1 band as 5–7 Hz and the lower α2 band as 7–9 Hz, whereas other studies (Hogan et al. 2003; Jelles et al. 2008) defined the upper α band as 9–11 Hz, with another study (Pijnenburga et al. 2004) defining the α band as 10–12 Hz. In our study, we did not divide α band into lower α1, lower α2, and upper α bands; we defined α band as 7–13 Hz.

We also found that the β band can reflect the differences between the younger group and the elderly group at the frontal, central, and parietal lobes. However, the β band was able to reflect the differences only between MCI patients and normal elderly group in the T3–TP7 and C3–CP3 electrode pairs. Although the CMI average data did not show significant differences between the elderly and MCI groups in the β band, the decreasing trends of aging and disease are consistent with previous studies (Pijnenburga et al. 2004; Jelles et al. 2008). Moreover, the results showed that the γ band could not reflected the differences between age-related changes and disease-related changes.

## Conclusions

In conclusion, this study showed that δ, θ, α, and β bands by CMI analysis were affected by age-related changes. In δ and θ bands, the corticocortical connections were shown on the parietal and occipital lobe; in α band, the corticocortical connections were shown almost on the whole brain; in β band, the corticocortical connections were shown on the frontal, central, and parietal lobe. Oppositely, only θ band by CMI analysis was affected by disease-related changes, especially in the corticocortical relationship on the parietal lobe. This study was subject to a limitation. Due to the relatively small sample size in this study, these results should, perhaps, be interpreted with caution. To sum up, this study found that only θ band can reflect the differences both of the age- and disease-related featured.
